# 

**Published:** 1874-10

**Authors:** 


### TWELFTH ANNUAL MEETING OF THE CONNECTICUT VALLEY DENTAL SOCIETY.

This Society will be held at Haynes' Hotel, Springfield, Mass., Tuesday and Wednesday, Oct. 13th and 14th, 1874.

ORDER OF BUSINESS.

Reading of Records.
Admission of New Members.
Report of Treasurer.
Election of Officers.
Retiring President's Address.
Induction of Officers.

The Executive Committee present the following subjects for discussion, and invite members to prepare papers upon them:

1. Irregularity and its treatment.
2. Prevention and cure of proximal caries.
3. Riggs' Disease, its diagnosis and treatment.
4. Gold foil, its essential properties, and instruments adapted to obtain the best results.
5. Hæmorrhage after extraction.
6. New Dental Appliance. Should old or new methods be universally adopted?
7. Artificial Dentures.
8. Miscellaneous Subjects.

Members of the profession are invited to bring their pet instruments; and proprietors of Dental Depots to exhibit any new dental appliance, or instruments, and explain their use under article Sixth, Wednesday, a. m.

OFFICERS:

President, H. M. Miller;
1st Vice-President, L. Parmelee;
2d Vice-President, J. J. Anderson;
Sec., L. C. Taylor;
Treasurer, E. M. Goodrich.

EXECUTIVE COMMITTEE:

H. F. Bishop, J. S. Hurlbut, O. R. Post.

L. C. TAYLOR, Secy.

### CONTENTS:

### COMMUNICATIONS:

### THE DENTAL REGISTER,

### A Monthly Magazine devoted entirely to the Interest of

### THE DENTAL PROFESSION.

**Terms Reduced to $2 50 per Year. Single Numbers 25cts.**

**EDITED BY PROF. J TAFT,**

**And Published by SPENCER CROCKER & CO., Ohio Dental Depot.**

The volume commences in January and closes in December.

The REGISTER being issued monthly is always fresh, therefore indispensable to the practitioner who desires to keep posted up with the new inventions and improvements which are daily developed. Neither expense nor effort will be spared to give value and variety to its contents. Articles will be illustrated with well executed engravings whenever they will add to the interest or assist in explanation.

Its extensive circulation and frequency of issue make it one of the BEST DENTAL ADVERTISING MEDIUMS in the United States.

All subscriptions must begin with January or July.

### RATES OF ADVERTISING.

| | 1 Mo. | 3 Mo. | 6 Mo. | 1 Year. | | 1 Year. | |
|---|---|---|---|---|---|---|---|
| 1 page.... | $12 00 | $25 00 | $40 00 | $75 00 | Inserted pages must be plain paper and black ink. | 1st page. | $50 00 |
| ½ page ... | 8 00 | 15 00 | 25 00 | 40 00 | | 2d page. | 37 50 |
| ¼ page ... | 5 00 | 10 00 | 15 00 | 25 00 | | 3d page. | 33 33 |
| | | | | | | 4th page. | 25 00 |

Local or special notices, five lines or less, will be inserted for $3 each insertion: over five lines, 50 cts. per line

All advertising bills payable in advance, or at furthest, after the issue of the first number containing the advertisement. All transient advertisements to be paid for in advance.

☞ CONTRIBUTIONS SOLICITED.

Exclusively Editorial Communications should be addressed to

**DR. J. TAFT, EDITOR.**

The latest number of the REGISTER may be ordered with or without other goods at any time, and all letters should be addressed to

**SPENCER, CROCKER & CO., OHIO DENTAL DEPOT,**
Cincinnati, Ohio.

